# Kinetically Controlled Direct Synthesis of Ag Nanoclusters as Precursor of Luminescent AgAu Alloy Nanoclusters for Aluminum Ions Detection

**DOI:** 10.3390/nano14241987

**Published:** 2024-12-12

**Authors:** Xianhu Liu, Yanping Chang, Wanqing Yao, Long Li, Hongwei Guo

**Affiliations:** Department of Chemistry and Environment, Jiaying University, Meisong Road 100, Meizhou 514015, China

**Keywords:** kinetically controlled synthesis, silver nanoclusters, silver–gold alloy nanoclusters, aluminum ions, aggregation-induced emission enhancement

## Abstract

Direct preparation of silver nanoclusters is of great significance for their applications. In this work, by selecting sodium cyanoborohydride as a weak reducing agent to control the kinetics of the reduction reaction, we successfully prepared silver nanoclusters protected by thiol-containing ligands, including mercaptosuccinic acid, cysteine, and glutathione. Based on the silver nanoclusters protected by mercaptosuccinic acid, silver–gold alloy nanoclusters were obtained through a gold doping reaction. Spectroscopic and particle size analyses showed that the silver–gold alloy nanoclusters exhibited aggregation-induced emission enhancement (AIEE) properties. A fluorescent probe for aluminum ions was developed based on the silver–gold alloy nanoclusters. In the presence of methionine and mercaptoacetic acid, the probe demonstrated good selectivity for aluminum ion detection. The linear range of this detection method was 0 to 192 μM, with a detection limit of 1.6 μM. The working mechanism of this detection method was further investigated through spectroscopic analysis.

## 1. Introduction

Metal nanoclusters, distinguished by their ultra-small size, uniform composition, and ease of surface functionalization, exhibit broad potential for applications in catalysis, optoelectronics, imaging, and sensing [1,2,3,4,5,6]. As the foundation for their applications, the preparation methods of metal nanoclusters have garnered considerable interest from researchers. Notably, the star Au_25_ nanocluster was successfully synthesized utilizing the Brust–Schiffrin two-phase synthesis approach [7]. By adopting the ligand exchange method, structural transformation of nanoclusters can be achieved, facilitating the tuning of their functionalities [8]. Additionally, the Galvanic/anti-Galvanic reaction [9] and intercluster reactions [10] among different types of nanoclusters provide avenues for the synthesis of doped metal nanoclusters with differing metal types and atomic ratios. Furthermore, etching methods enable the production of relatively stable nanoclusters [11]. Solid-phase synthesis presents a green and facile method for synthesizing metal nanoclusters [12].

In some of the aforementioned preparation methods, modulating the kinetics of the reduction reaction has proven to promote product purity and boost ultimate yields. This method avoids complex multi-step reaction processes, facilitating the direct synthesis of metal nanoclusters. For example, by decreasing the reaction temperature and agitation speed, high-purity oil-soluble Au_25_ nanoclusters can be synthesized [7]. Adjusting the pH of the solution with sodium hydroxide to control the reducing power of sodium borohydride also allows for the preparation of high-purity water-soluble Au_25_ nanoclusters [13]. By opting for carbon monoxide as a gaseous reductant, researchers can discern the entire formation process of water-soluble Au_25_ nanoclusters [14]. Utilizing tert-butylammonium borane as a weak reductant, the successful synthesis of Au_15_ and Au_18_ nanoclusters has been demonstrated [15]. Similarly, employing sodium cyanoborohydride as a weak reductant has led to the successful synthesis of Au_18_ and Au_49_ nanoclusters [16,17].

Aluminum, which finds extensive applications in industrial production and daily life, has garnered increasing attention regarding its potential toxicity [18,19,20]. Accumulation of excessive aluminum in the human body can result in diseases such as Alzheimer’s and Parkinson’s. The World Health Organization (WHO) has established stringent guidelines for the aluminum content in drinking water and daily human intake [21,22]. Furthermore, excessive aluminum in the environment can adversely impact soil microstructure and plant growth [23,24]. The detection of aluminum ions typically relies on sophisticated instruments such as inductively coupled plasma mass spectrometry (ICP-MS), atomic absorption spectrometry, and atomic emission spectrometry [25,26,27]. However, these methods often entail rigorous sample pretreatment and complex testing procedures. Fluorescence detection methods offer the advantages of high sensitivity and streamlined testing. As a result, aluminum ion probes based on fluorescence detection principles have emerged as a research focus. For example, small-molecule fluorescence probes derived from naphthalene derivatives and salicylaldehyde acyl-hydrazone compounds have been employed for detecting aluminum ions in aqueous solutions [28,29]. Ratiometric fluorescence probes for aluminum ion detection have been developed based on the excited-state intramolecular proton transfer (ESIPT) principle [30]. By utilizing esterification reactions on ethylcellulose, researchers have successfully synthesized flavonol derivative-based fluorescence probes for aluminum ions [31]. In recent years, the use of fluorescent nanomaterials for the detection and analysis of aluminum ions has garnered significant attention. ZnS:Mn nanoparticles functionalized with morin and tetracycline-based carbon dot nanomaterials have been applied as fluorescence probes for aluminum ions [32,33]. Fluorescent gold–silver nanoclusters can be used to analyze the concentration of aluminum ions in aqueous solutions via the mechanism of aggregation-induced emission enhancement [34,35]. Furthermore, gold-doped copper nanoclusters exhibiting aggregation-induced emission properties can be utilized for the simultaneous detection of aluminum ions and norfloxacin [36].

In this research work, we chose the mild reducing agent sodium cyanoborohydride for the preparation of silver nanoclusters protected by single-ligand and mixed-ligand-protected. Using the silver nanocluster as precursor, silver–gold alloy nanoclusters were further synthesized through gold doping. Experiments revealed that the silver–gold alloy nanoclusters exhibited aggregation-induced emission enhancement (AIEE) characteristics. Based on the silver–gold alloy nanoclusters, we developed a fluorescence-enhanced probe for aluminum ions. The probe demonstrated good selectivity and sensitivity for detection, with a linear detection range of 0 to 192 μM and a limit of detection of 1.6 μM. Experimental results indicated that the mechanism of this fluorescence probe for aluminum ion detection was based on the aggregation-induced fluorescence enhancement phenomenon.

## 2. Materials and Methods

### 2.1. Synthesis of Ag NCs and Luminescent AgAu NCs

Silver sulfate (9 mg) was completely dissolved in ultrapure water (2 mL) under ultrasonic treatment. Mercaptosuccinic acid (18 mg) was added for complexing with silver ions and the reacting process lasted for 15 min under stirring at room temperature. Then, 1 mL of sodium cyanoborohydride aqueous solution (19 mg/mL) was added dropwise to a reduction of silver thiolate under stirring. The solution was centrifuged at 8000 rpm for 3 min after formation of the silver nanocluster. The supernatant was mixed with excess methanol to precipitate the silver nanocluster. The solid product was rinsed with methanol three times via ultrasonic treatment and centrifugation. Drying of the above product in a vacuum chamber could give the powder of final Ag NC-1. The preparation methods of Ag NC-2 and Ag NC-3 were similar to that of Ag NC-1, except the difference in types and quantities of ligands. In the case of Ag NC-2, the procedure involved incorporating 19 mg of glutathione and 7 mg of cysteine. For the synthesis of Ag NC-3, the ligands consisted of 19 mg of glutathione and 9 mg of mercaptosuccinic acid.

The Ag NC-1 (5 mg) was dissolved in ultrapure water (1 mL) assisted by ultrasonic treatment. Then, 80 μL of chloroauric acid aqueous solution (45 mg/mL) was added. After stirring for 2 h at room temperature, the solution was centrifuged at 8000 rpm for 3 min. The supernatant containing AgAu NCs was used as stock solution which was preserved away from light at room temperature. The reacting time and ratios of reactants were optimized to enhance the luminescent intensity of products. The reacting time ranged from 20 to 240 min and the volumes of chloroauric acid aqueous solution (45 mg/mL) changed within the range of 70 to 100 μL.

### 2.2. Aluminum Ion Detection

Thirty microliter metal ions (Fe^3+^, Fe^2+^, Ni^2+^, Zn^2+^, Cd^2+^, Mn^2+^, Cr^3+^, Pb^2+^, Cu^2+^, Co^2+^, Ca^2+^, Al^3+^, Ag^+^, Na^+^, Mg^2+^) stock solution (10 mM), 2.87 mL ultrapure water, and 100 μL AgAu NCs stock solution were mixed uniformly by shaking. The fluorescence spectra were recorded from 500 to 800 nm with the maximum excitation wavelength at 450 nm. The competition experiments were conducted via replacing the single metal ion with mixed metal ions and the volume of metal ions solution remained unchanged. Mercaptoacetic acid (50 μL, 10 mmol/L) and methionine (0.5 mL, 10 mmol/L) were added to the above mixed solution to improve selectivity of aluminum ion analysis. Different volumes of aluminum ion stock solution (10 mM) were mixed with 2.2 mL ultrapure water and 0.3 mL AgAu NCs stock solution for the quantitative analysis of the fluorescent probe.

## 3. Results and Discussion

### 3.1. Synthesis and Characterization of the Ag NCs and AgAu NCs

**Figure 1:**
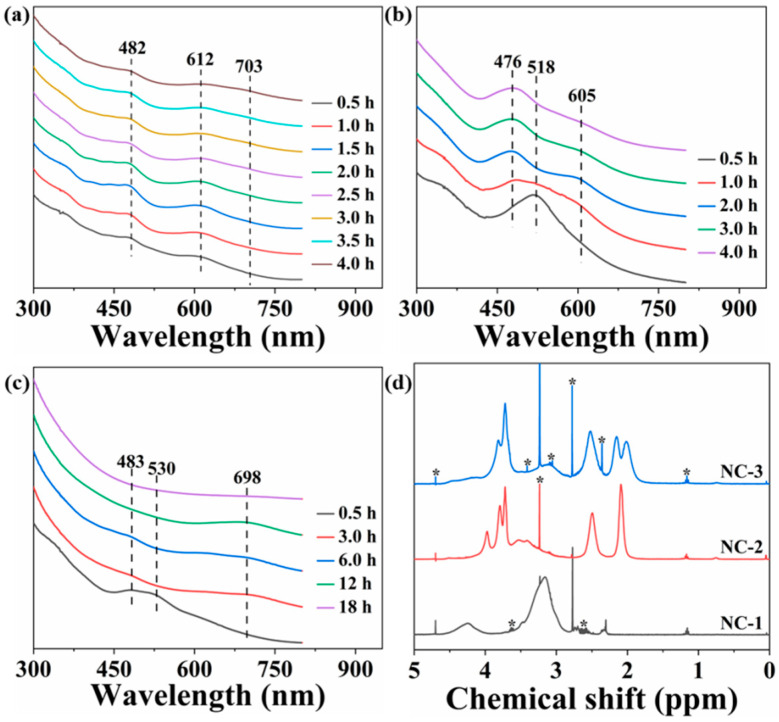
UV-Vis absorption spectra of Ag NC-1 (**a**), Ag NC-2 (**b**), and Ag NC-3 (**c**) at different reaction times. ^1^H-NMR spectra of the prepared Ag NCs (**d**). The peaks marked with an asterisk correspond to the solvent signals.

This study delved into using the weak reducing agent sodium cyanoborohydride to control the reaction kinetics, aiming to directly synthesize silver nanoclusters. Firstly, mercaptosuccinic acid (MSA) was opted for synthesizing silver nanoclusters (Ag NC-1). Within 0.5 h of the reaction, distinct absorption peaks emerged at 482 and 612 nm in the UV-Vis absorption spectrum (Figure 1a). As the reaction progressed, a novel absorption peak at 703 nm could be observed. These absorption peaks significantly differed from reported spectral features of silver nanoclusters [37,38,39,40], suggesting the formation of a unique type of silver nanocluster. Subsequently, we investigated the feasibility of this method for synthesizing silver nanoclusters protected by mixed ligands. When cysteine (CYS) and glutathione (GLU) were utilized as ligands to synthesize silver nanoclusters (Ag NC-2), an initial UV-Vis absorption peak was observed at 518 nm (Figure 1b). However, upon reaching 2 h of reaction time, this peak vanished, and two new peaks emerged at 476 and 605 nm. Alternatively, when glutathione and mercaptosuccinic acid were combined as mixed ligands for synthesizing silver nanoclusters (Ag NC-3), initial absorption peaks were detected at 483 and 530 nm. After 12 h of reaction (Figure 1c), these peaks disappeared, replaced by a single peak at 698 nm. The molecular formulas of Ag NC-1, Ag NC-2, and Ag NC-3 were determined to be Ag_14_(MSA)_8_, Ag_10_(GLU)_6_(CYS)_2_, and Ag_11_(GLU)_5_(MSA)_1_ based on the results of ESI mass spectra (Figures S1–S3). It should be emphasized that these assignments necessitate further verification through single-crystal X-ray diffraction analysis. However, we have been unsuccessful in growing single crystals of these nanoclusters. The mean particle diameters of Ag NC-1, Ag NC-2, and Ag NC-3 were 2.3 nm, 3.5 nm, and 2.1 nm (Figures S4–S6), respectively. It is worth highlighting that the actual size distribution may be even more confined than displayed in the TEM images, given that nanoclusters tend to aggregate during the TEM measurement. To further elucidate the surface chemistry, ^1^H-NMR spectroscopy of the prepared silver nanoclusters was performed (Figure 1d and Figures S7–S9). The ^1^H-NMR spectrum of Ag NC-1 predominantly exhibited signals attributed to mercaptosuccinic acid, whereas Ag NC-2 and Ag NC-3 displayed signals corresponding to mixed ligands’ composition of cysteine, glutathione, and mercaptosuccinic acid. These results affirmed the successful bonding of the introduced ligands on the surface of the synthesized silver nanoclusters.

**Figure 2:**
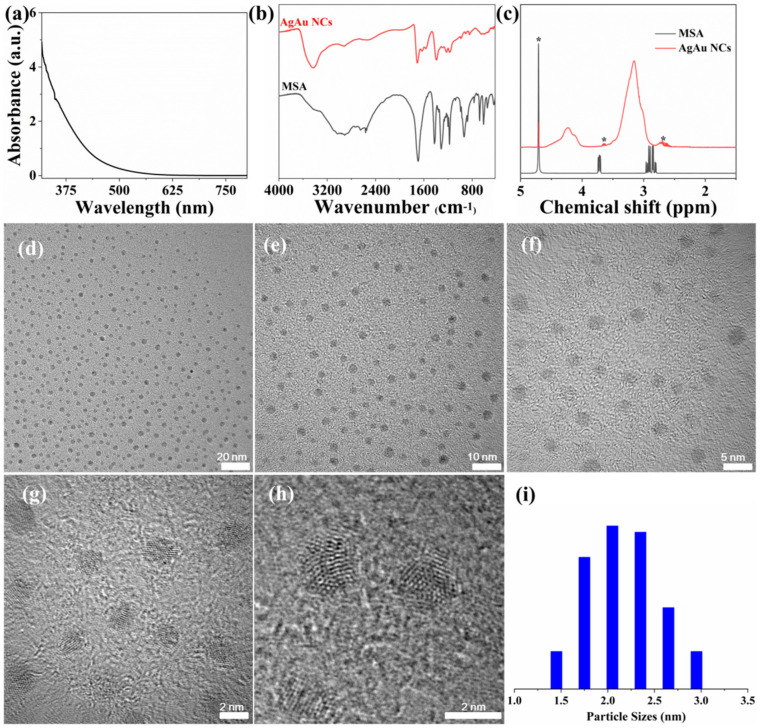
(**a**) UV-Vis absorption spectrum of AgAu alloy nanoclusters. (**b**) Infrared absorption spectra and (**c**) ^1^H NMR spectra of AgAu alloy nanoclusters and mercaptosuccinic acid. The peaks marked with an asterisk correspond to the solvent signals. (**d**–**i**) TEM pictures and statistical analysis of the sizes of AgAu alloy nanoclusters.

Subsequently, the prepared Ag NC-1 served as precursor to synthesize AgAu alloy nanoclusters with chloroauric acid as reactant. In comparison to previously reported methods for synthesizing AgAu alloy nanoclusters, the preparation method employed in this study offers several advantages. The precursor silver nanoclusters can be synthesized through a kinetically controlled direct method, ensuring ease of availability. The synthesis process can be carried out at room temperature, negating the requirement for maintaining elevated or cryogenic conditions. Furthermore, the preparation time is limited to just 2 h, marking a reduction in duration. In the UV-Vis absorption spectrum of the prepared AgAu alloy nanoclusters (Figure 2a), no distinct characteristic absorption peaks were observed, suggesting that the product likely contained a mixture of alloy nanoclusters with varying sizes. Through mass spectrometry characterization (Figure S10), it was found that the main component in the mixture was Ag_7_Au_4_(MSA)_11_. Infrared spectroscopy was utilized to characterize the AgAu alloy nanoclusters and ligand mercaptosuccinic acid (Figure 2b). In comparison, the absorption peaks of ligand were also observable in the spectrum of AgAu alloy nanoclusters. However, these absorption peaks exhibited shifts and an increase in half-peak width compared with those of mercaptosuccinic acid. According to the previous reports, it was speculated that this phenomenon was caused by electron transfer between metals and ligands, as well as spatial interactions within the nanoclusters. Notably, the absorption peak at 2530 cm^−1^ in the spectrum of mercaptosuccinic acid, corresponding to the stretching vibration of -SH, disappeared in the spectrum of AgAu alloy nanoclusters, suggesting that the reaction between ligand and metal primarily occurred through thiol groups [34]. Additionally, ^1^H-NMR spectroscopy was employed to analyze the surface functional groups of the AgAu alloy nanoclusters (Figure 2c). The test results indicated that the ^1^H-NMR signals corresponding to methylene and methine groups in mercaptosuccinic acid also exhibited shifts and broadening in the spectrum of AgAu alloy nanoclusters. Apart from the factors discussed above, such as electron transfer and spatial interactions, this phenomenon might also be attributed to the presence of multiple ligands on the surface of nanocluster, each residing in a unique chemical environment. Transmission electron microscopy (TEM) was utilized to characterize the particle sizes of the AgAu alloy nanoclusters (Figure 2d–i). A statistical analysis of the sizes of about 200 particles revealed that the nanoclusters had diameters ranging from 1.5 to 3.0 nm, with an average diameter of 2.2 nm.

**Figure 3:**
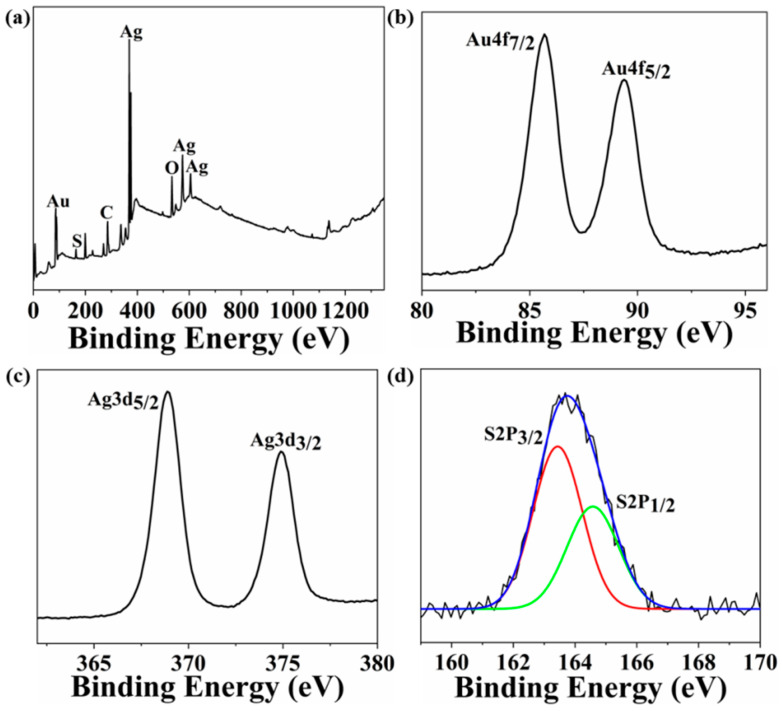
(**a**) XPS survey spectrum of AgAu alloy nanoclusters. (**b**–**d**) The high-resolution spectra analysis of gold, silver, and sulfur elements in AgAu alloy nanoclusters.

X-ray photoelectron spectroscopy (XPS) was employed to investigate the elemental composition and valence state of the elements of AgAu alloy nanoclusters. The survey spectrum results demonstrated that the AgAu alloy nanoclusters were comprised of C, O, S, Ag, and Au elements (Figure 3a), which were in accordance with the elemental composition of ligand and metal reactants, confirming that the final product was the AgAu alloy nanoclusters protected by mercaptosuccinic acid. The high-resolution spectrum analysis of gold element revealed that the electron binding energy of Au 4f_7/2_ was 85.7 eV, within the range between Au(0) (84 eV) and Au(I) (86 eV) (Figure 3b), indicating that the gold element in the AgAu alloy nanoclusters existed in both zero valence and monovalence forms. The electron binding energy of Ag 3d_5/2_ was 368.9 eV, which was greater than that of Ag(I) (368.2 eV) (Figure 3c), suggesting that the silver element was predominantly present in the monovalence state. Furthermore, the high-resolution test result for sulfur element indicated that the electron binding energy of S 2P_3/2_ was 163.4 eV (Figure 3d), which was close to the binding energy of reported metal sulfides [40].

### 3.2. Luminescent Properties of the AgAu NCs

**Figure 4:**
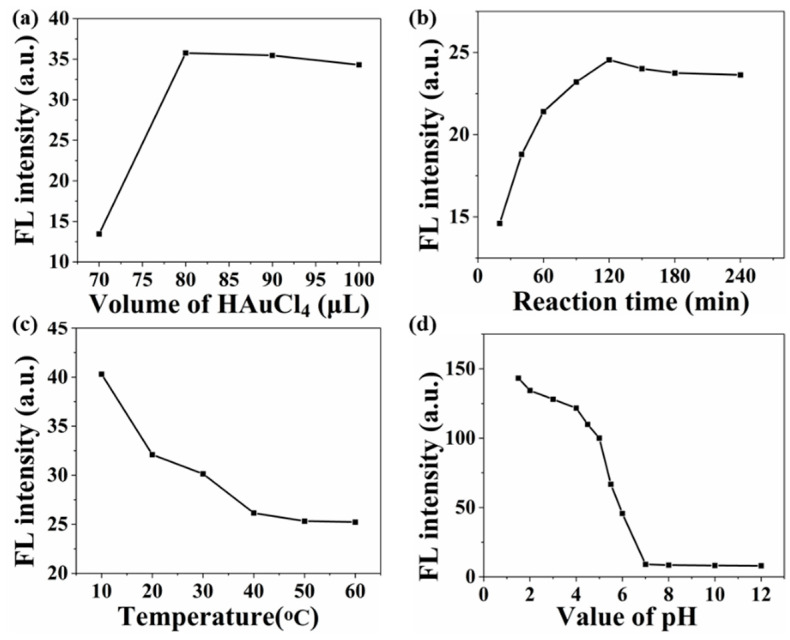
The influences of quantities of added chloroauric acid (**a**), reaction time (**b**), temperature (**c**), and pH values (**d**) for the fluorescence intensity of the AgAu alloy nanoclusters.

The AgAu alloy nanoclusters exhibited red fluorescence when excited by ultraviolet light. Fluorescence spectroscopy characterization revealed that the maximum excitation and emission wavelengths of the AgAu alloy nanoclusters were approximately 450 nm and 700 nm (Figures S11 and S12), respectively. The reactant ratios and reaction time were optimized to improve the fluorescence property of the AgAu alloy nanoclusters (Figure 4a,b). It was discovered that the fluorescence reached maximum intensity when the mass ratio of chloroauric acid to silver nanoclusters was 0.72 and the reaction time was 2 h. Additionally, the influences of external environmental factors on the fluorescence intensity of the AgAu alloy nanoclusters were examined. The fluorescence intensity was negatively correlated with temperature (Figure 4c), which was attributed to the fact that rising temperatures could increase vibration frequency of the functional groups and structures of nanoclusters, thereby enhancing the probability of non-radiative transitions of the excited state. As the pH value of the aqueous solution increased, the fluorescence intensity of the AgAu alloy nanoclusters also decreased (Figure 4d). Notably, when the solution pH approached neutrality, the fluorescence intensity rapidly diminished. In alkaline solutions, the AgAu alloy nanoclusters were almost non-luminescent. Furthermore, the effects of various solvents on the luminescence intensity were explored (Figure S13). It was observed that the AgAu alloy nanoclusters displayed significant fluorescence enhancement in ethanol and methanol in comparison with their fluorescence intensity in water.

**Figure 5:**
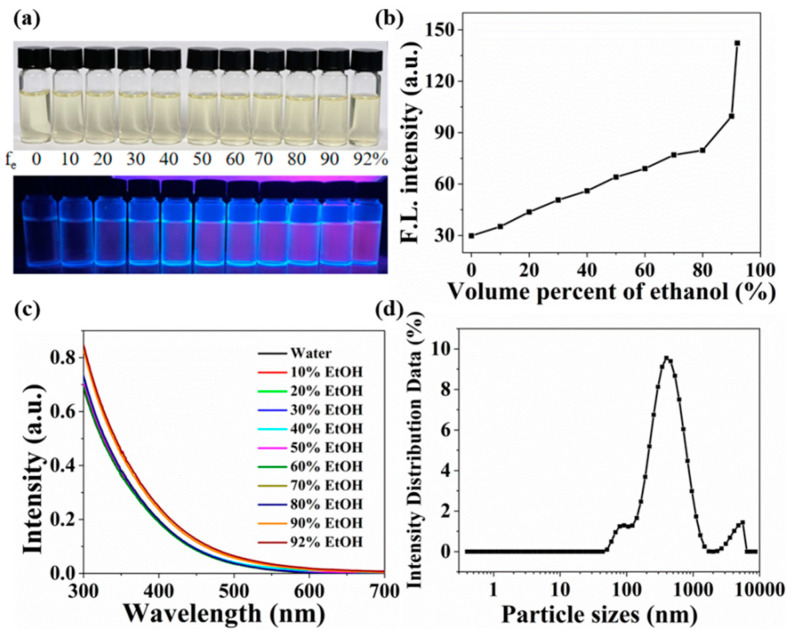
(**a**) Digital photos of AgAu alloy nanoclusters in mixed solvents of ethanol and water with different volume fractions under visible (top row) and UV (bottom row) light. Emission intensity (**b**) and UV-Vis absorption spectra (**c**) of AgAu alloy nanoclusters in mixed solvents of ethanol and water with different volume fractions. (**d**) Dynamic light scattering spectra of AgAu alloy nanoclusters dispersed in 92% ethanol.

During investigation of the influence of the solution pH on luminescence intensity of the AgAu alloy nanoclusters, it was observed that when the pH value was low, the solution became turbid, accompanied by a notable increase in fluorescence intensity. Similarly, when the nanoclusters were dispersed in ethanol and methanol, turbid solutions were formed, exhibiting significantly enhanced fluorescence. Based on these findings, it was inferred that the AgAu alloy nanoclusters possessed the characteristic of aggregation-induced emission enhancement (AIEE). To further validate this characteristic, the nanoclusters were dispersed in a mixed solvent of water and ethanol (Figure 5a), and their luminescence and UV-Vis absorption were analyzed. The experimental results revealed a gradual increase in fluorescence intensity as the ethanol content in the mixed solvent increased (Figure 5b). According to the UV-Vis absorption spectra of the solutions (Figure 5c), an overall increase in absorption was observed within the range of 300 to 600 nm when the ethanol content reached 90% and 92%. This was attributed to the prominent aggregation of nanoclusters in the solution as the ethanol content exceeded 90%, leading to the significant scattering effect [41]. Furthermore, laser dynamic light scattering analysis of the alloy nanoclusters solution containing 92% ethanol showed that the particle size reached 369 nm (Figure 5d), significantly larger than the particle size of the alloy nanoclusters. Thus, the above results demonstrated that the AgAu alloy nanoclusters exhibited the characteristic of AIEE.

### 3.3. Aluminum Ion Detection

**Figure 6:**
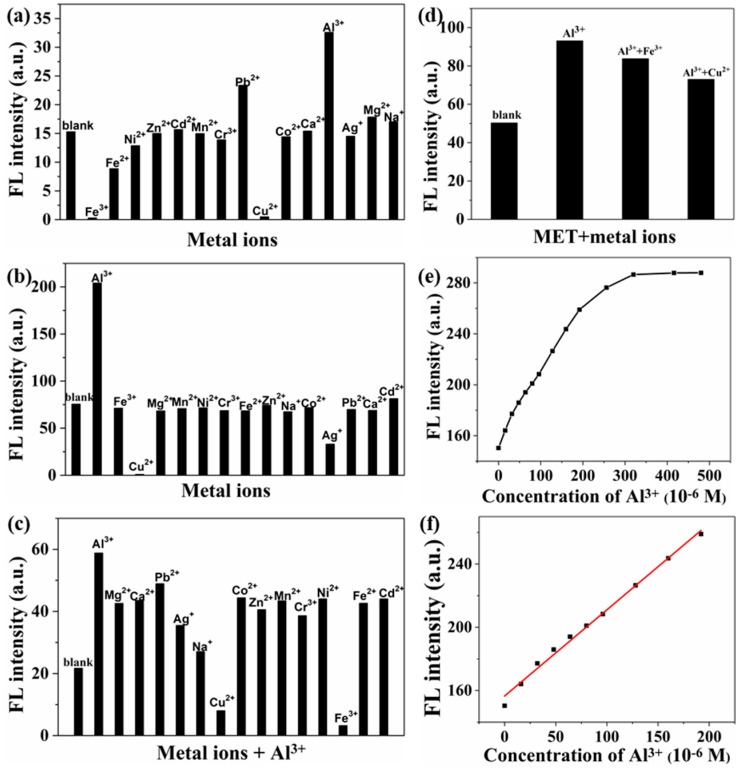
The fluorescence variation of AgAu alloy nanoclusters in the presence of metal ions (**a**), metal ions+mercaptoacetic acid (**b**), metal ions+Al^3+^ ions (**c**), and metal ions+methionine (**d**). (**e**) Fluorescence intensity changes of AgAu alloy nanoclusters as a function of concentrations of Al^3+^ ions. (**f**) The Stern–Volmer plot showing the changes of fluorescence intensity of AgAu alloy nanoclusters corresponding to varying concentrations of Al^3+^ ions in the range of 0 to 192 μM.

The fluorescent AgAu alloy nanoclusters were explored for the detection of metal ions in aqueous solutions. Compared to ZnS:Mn nanoparticles functionalized with morin and tetracycline-based carbon dot [32,33], the aluminum ions probe based on AgAu alloy nanoclusters exhibits the following advantages. The probe preparation process is simplified and does not require any post-modification steps. The probe preparation can be completed at room temperature without the need for heating. By mixing different types of metal ions with AgAu alloy nanoclusters, the fluorescence spectroscopy was employed to analyze the influence of metal ions on the fluorescence intensity of nanoclusters (Figure 6a). The results indicated that aluminum ions and lead ions significantly enhanced the emission of the nanoclusters, whereas iron ions and copper ions considerably diminished their fluorescence intensity. To utilize AgAu alloy nanoclusters for aluminum ion detection, mercaptoacetic acid was firstly used as a masking reagent to eliminate the interference of lead ions (Figure 6b). The results showed that after the addition of mercaptoacetic acid, the fluorescence intensity of the nanoclusters was virtually unaffected by lead ions. Additionally, during competitive experiments, it was observed that when iron ions or copper ions coexisted with aluminum ions (Figure 6c), the fluorescence of the nanoclusters remained decreased, suggesting that iron ions and copper ions also interfered with aluminum ions detection. To eliminate the interference, another masking reagent methionine was introduced (Figure 6d). The results demonstrated that methionine effectively eliminated the interference of iron ions and copper ions, enabling AgAu alloy nanoclusters to be applied for the selective detection of aluminum ions.

Next, the AgAu alloy nanoclusters were investigated for the quantitative analysis of aluminum ions concentration in aqueous solutions. Experimental results indicated that as the concentrations of aluminum ions continually increased, the fluorescence intensity of AgAu alloy nanoclusters exhibited a corresponding gradual increase (Figure 6e). When the aluminum ions concentration reached 287 μM, the fluorescence of the clusters remained unchanged. Further analysis revealed that within the range of 0 to 192 μM, there was a good linear relationship between the aluminum ions concentration and the fluorescence intensity of the nanoclusters (Figure 6f), represented by the linear equation y = 156 + 0.55x, where y denoted the fluorescence intensity and x represented the aluminum ions concentration. The correlation coefficient R^2^ of this equation was 0.992. Based on this linear equation and using a signal-to-noise ratio of three as the basis for calculation, the limit of detection for quantitative analysis of aluminum ions was determined to be 1.6 μM.

**Figure 7:**
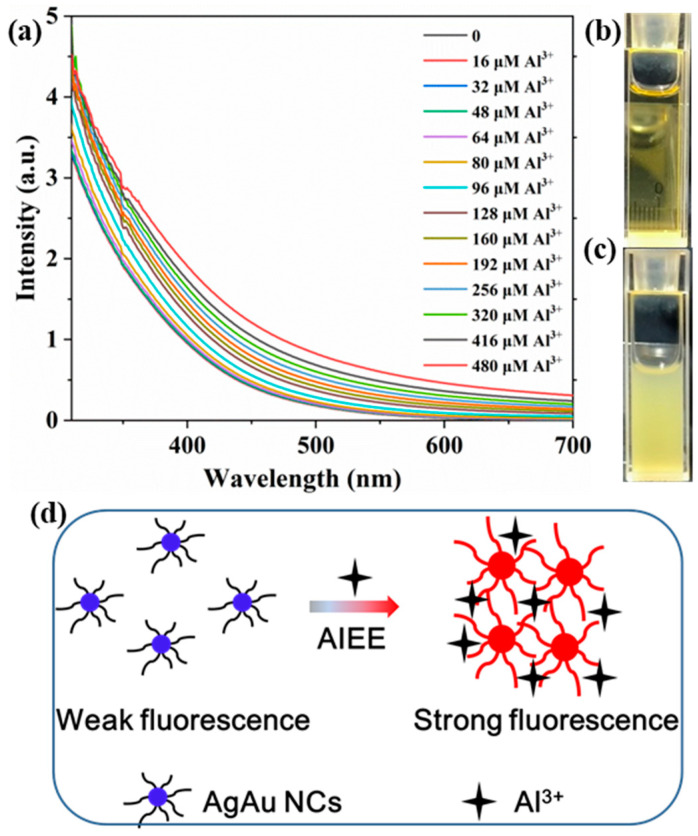
(**a**) UV-Vis absorption spectra of AgAu alloy nanoclusters in solutions with different concentrations of aluminum ions. Photographs of AgAu alloy nanoclusters dispersed in water (**b**) and 480 μM aluminum ions solution (**c**). Scheme illustration for the detection mechanism of aluminum ions (**d**).

To further explore the mechanism of AgAu alloy nanoclusters in detecting aluminum ions, UV-Vis absorption spectroscopy was employed to analyze solutions of AgAu alloy nanoclusters with different concentrations of aluminum ions (Figure 7a). The test results revealed that as the aluminum ions concentration increased, the absorption curve of the mixed solution exhibited an overall upward trend. This phenomenon is similar to the trend observed when AgAu alloy nanoclusters were dispersed in mixed solvents containing different proportions of ethanol and water. This indicated that the aggregation of nanoclusters occurred, leading to the formation of larger particles in the solution, which subsequently triggered a scattering effect. By comparing photographs of the AgAu alloy nanoclusters solution before and after the addition of aluminum ions (Figure 7b,c), it became apparent that the addition of aluminum ions caused the solution to transform from clear to turbid. Thus, the aforementioned results suggested that the mechanism of emission enhancement of AgAu alloy nanoclusters by aluminum ions was the AIEE effect (Figure 7d).

## 4. Conclusions

This study further demonstrates the importance of controlling the kinetics of the reduction reaction process for the direct preparation of high-purity silver nanoclusters. A weak reducing agent, sodium cyanoborohydride, was selected to synthesize three types of silver nanoclusters protected by single and mixed ligands. Using the prepared silver nanocluster as precursors, silver–gold alloy nanoclusters with AIEE property were prepared through gold doping. These silver–gold alloy nanoclusters were used for selective detection of aluminum ions in aqueous solutions, with a linear range of 0 to 192 μM and a detection limit of 1.6 μM. Spectral analysis results indicated that the mechanism for aluminum ions detection by the silver–gold alloy nanoclusters was due to the AIEE phenomenon.

## Data Availability

Data are contained within the article.

## Supplementary Materials

The following supporting information can be downloaded at: https://www.mdpi.com/article/10.3390/nano14241987/s1, Figure S1: ESI mass spectrum of Ag NC-1; Figure S2: ESI mass spectrum of Ag NC-2; Figure S3: ESI mass spectrum of Ag NC-3; Figure S4: TEM picture and sizes distribution of Ag NC-1; Figure S5: TEM picture and sizes distribution of Ag NC-2; Figure S6: TEM picture and sizes distribution of Ag NC-3; Figure S7: ^1^H-NMR of mercaptosuccinic acid; Figure S8: ^1^H-NMR of glutathione; Figure S9: ^1^H-NMR of cysteine; Figure S10: ESI mass spectrum of AgAu alloy nanoclusters; Figure S11: excitation spectrum of AgAu alloy nanoclusters; Figure S12: emission spectrum of AgAu alloy nanoclusters; Figure S13: emission intensity of AgAu alloy nanoclusters in different solvents.
