# Supplementary material for: Kinetically Controlled Direct Synthesis of Ag Nanoclusters as Precursor of Luminescent AgAu Alloy Nanoclusters for Aluminum Ions Detection
Source: Nanomaterials (Basel). 2024 Dec 12;14(24):1987. doi: 10.3390/nano14241987 (PMC11728816; doi:10.3390/nano14241987)
Supplement: Supplementary file 1 [file nanomaterials-14-01987-s001.zip › nanomaterials-3328765-supplementary.pdf]

Supplementary Information

Kinetically Controlled Direct Synthesis of Ag Nanoclusters as Precursor of Luminescent AgAu Alloy Nanoclusters for Aluminium Ions Detection

Xianhu Liu, Yanping Chang, Wanqin Yao, Long Li and Hongwei Guo*

Department of Chemistry and Environment, Jiaying University, Meisong Road 100, Meizhou 514015, Guangdong, P. R. China

Corresponding author: Hongwei Guo

Email: guohw624@qq.com

1、Reagents

Silver sulfate (98%), mercaptosuccinic acid (98%), sodium cyanoborohydride (99.5%), glutathione (98%), cysteine (98%), chloroauric acid (99%), mercaptoacetic acid (99%) and methionine were purchased from Energy Chemical (Shanghai, China). Hydrochloric acid (HCl, A.R.), methanol (A.R.), ethanol (A.R.) and sodium hydroxide (NaOH, A.R.) were purchased from Sinopharm Chemical Reagent Co. Ltd. (Shanghai, China). Water utilized in all tests was ultrapure. All other reagents were used as received without further purification.

2、Characterization Methods

The UV-vis adsorption spectrum was recorded on an Agilent Cary 300 UV-Vis spectrophotometer. The luminescence spectra were performed using a PerkinElmer LS 55 fluorescence spectrometer. The size and morphology of the product was observed on JEOL-Model 2100F transmission electron microscopy (TEM) and (FEI Technai-F30, USA). A Niolet iN10 Fourier transform infrared spectrometer was used to record the Fourier transform infrared (FT-IR) spectra of Cu NC and ligand. Dynamic light scattering analysis was carried out on the Malvern Zetasizer Nano S90 apparatus. ^{1}H-NMR spectra were performed on Bruker AVANCE NEO Ascend Evo 400. X-ray photoelectron spectroscopy (XPS) was recorded on the Thermo Scientific ESCALab 250Xi using 200 W monochromated Al Kα radiation.

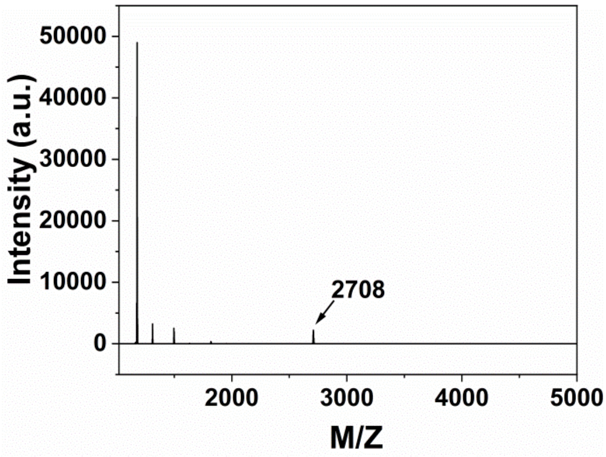

Figure S1. ESI mass spectrum of Ag NC-1.

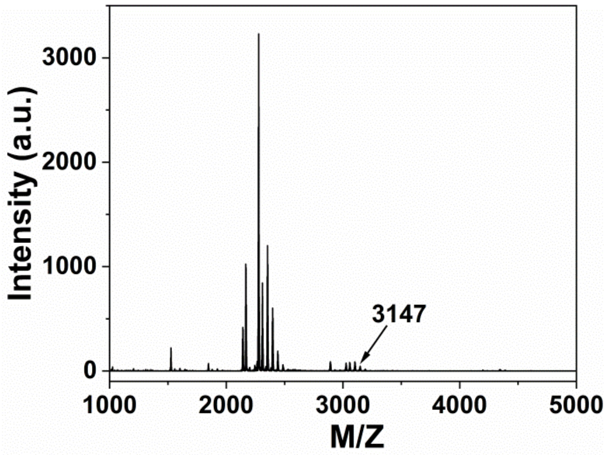

Figure S2. ESI mass spectrum of Ag NC-2.

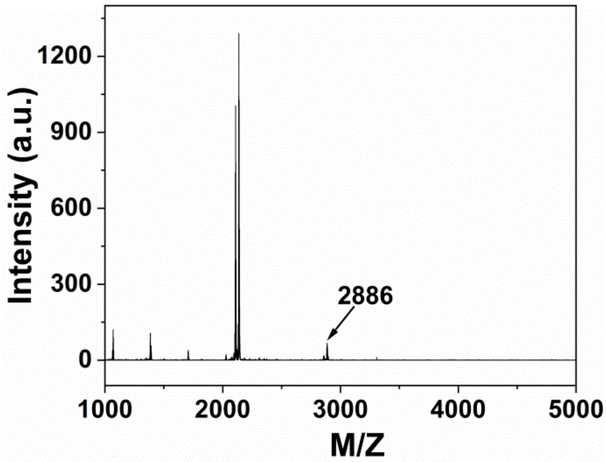

Figure S3. ESI mass spectrum of Ag NC-3.

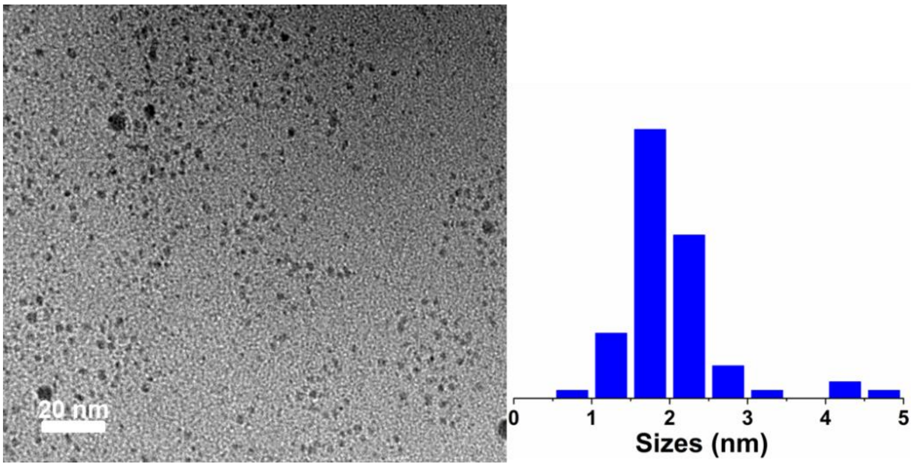

Figure S4. TEM picture and sizes distribution of Ag NC-1.

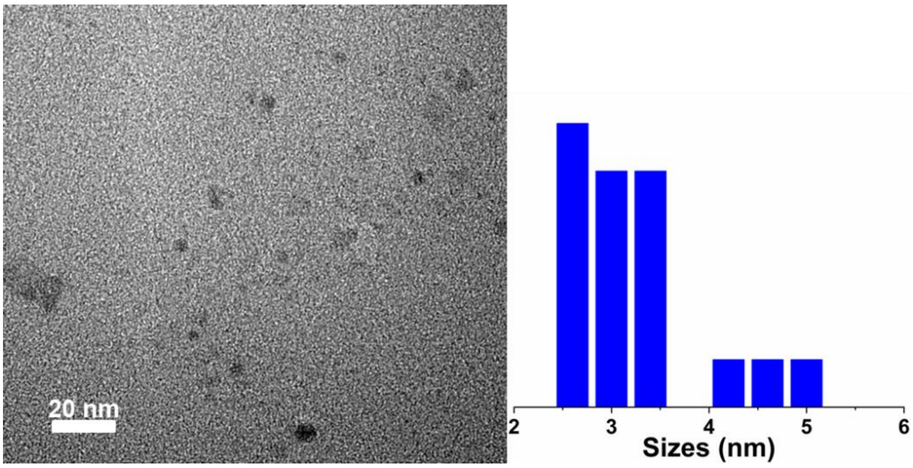

Figure S5. TEM picture and sizes distribution of Ag NC-2.

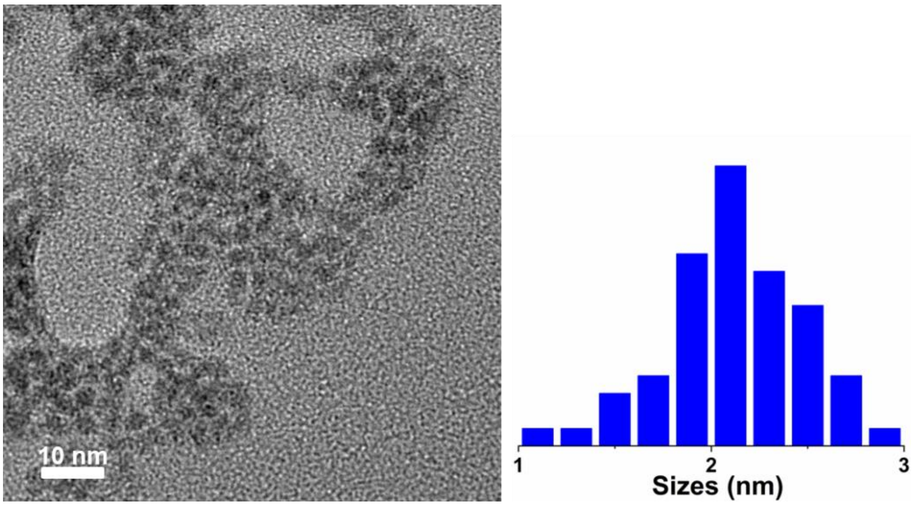

Figure S6. TEM picture and sizes distribution of Ag NC-3.

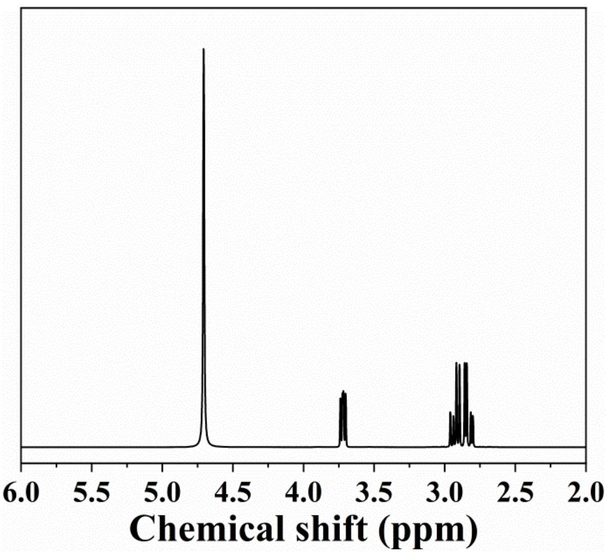

Figure S7. ^{1}H-NMR of mercaptosuccinic acid.

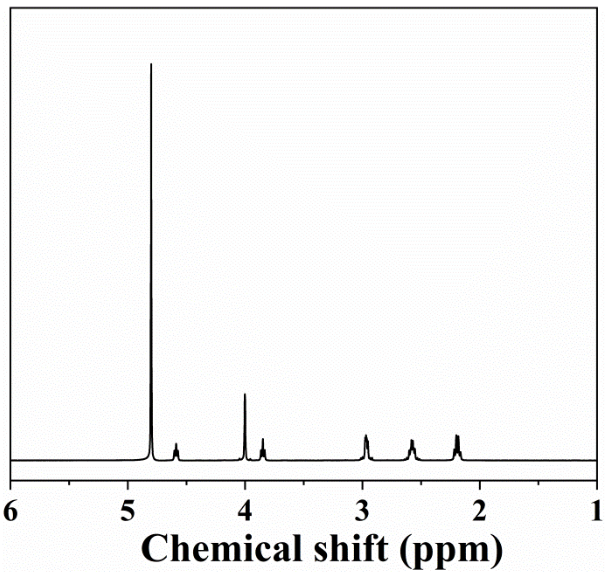

Figure S8. ^{1}H-NMR of glutathione.

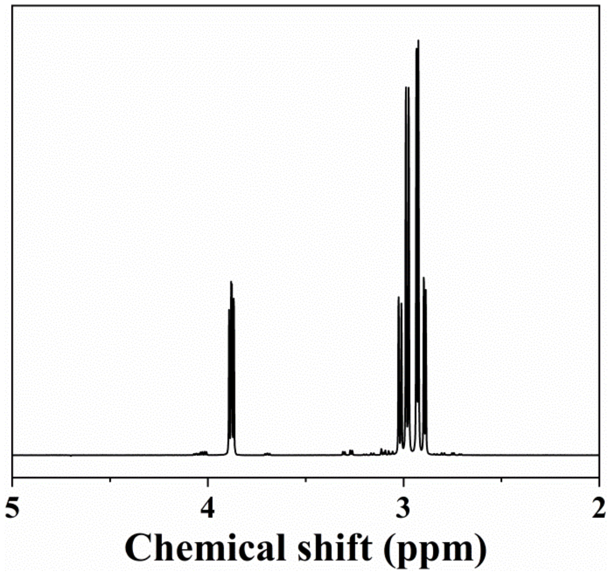

Figure S9. ^{1}H-NMR of cysteine.

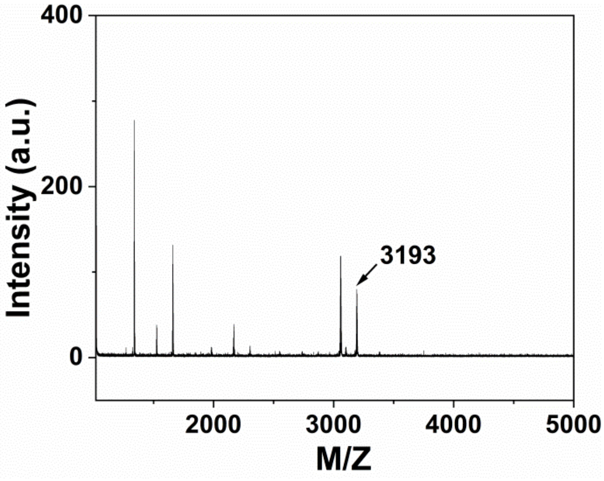

Figure S10. ESI mass spectrum of AgAu alloy nanoclusters.

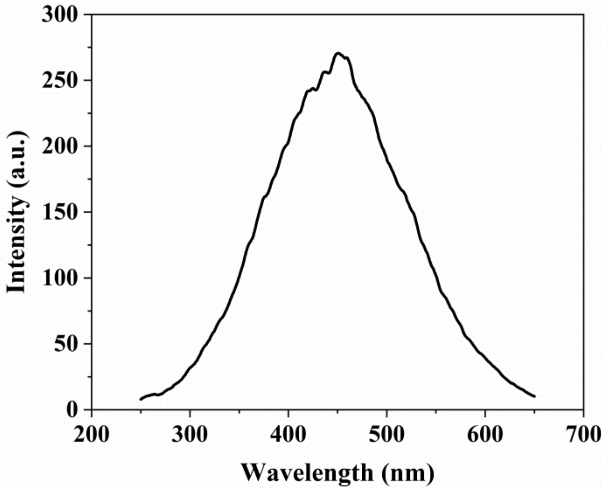

Figure S11. Excitation spectrum of AgAu alloy nanoclusters.

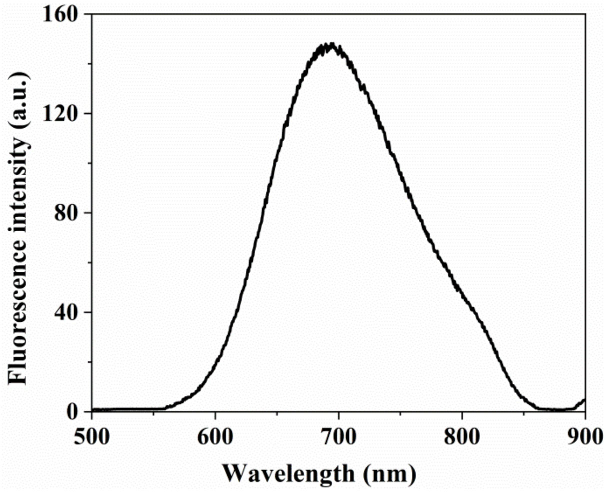

Figure S12. Emission spectrum of AgAu alloy nanoclusters.

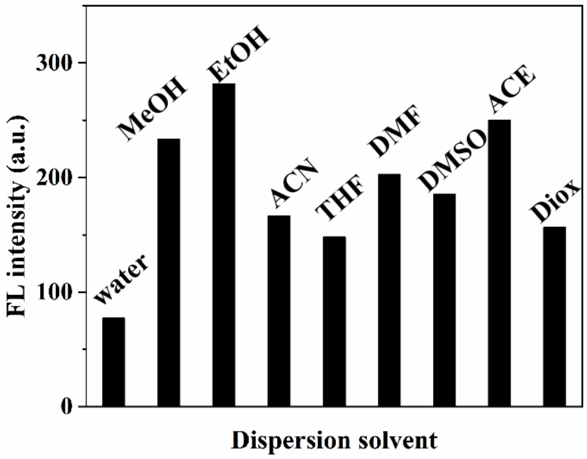

Figure S13. Emission intensity of AgAu alloy nanoclusters in different solvents.
